# Effective connectivity of the amygdala during the consumption of erotic, sexual humor, and monetary rewards with a DCM-PEB approach

**DOI:** 10.1371/journal.pone.0279281

**Published:** 2022-12-29

**Authors:** Yu-Chen Chan, Tai-Li Chou

**Affiliations:** 1 Department of Educational Psychology and Counseling, National Tsing Hua University, Hsinchu, Taiwan; 2 Department of Psychology, National Taiwan University, Taipei, Taiwan; UCSI: UCSI University, MALAYSIA

## Abstract

While a large body of research exists on the processing of monetary rewards, less is known about sexual reward processing. This study aimed to identify effective connectivity for the consumption of sexual (erotic and sexual humor) and non-sexual (monetary) rewards, using dynamic causal modeling and parametric empirical Bayes with subjective hedonic ratings included. Our results support the importance of the amygdala for sexual humor amusement, the nucleus accumbens (NAc) for monetary rewards, and the lateral orbitofrontal cortex (lOFC) for erotic pleasure. The amygdala, NAc, and lOFC are major dopaminergic targets with known roles in the reward circuitry. Appreciating sexual humor was associated with ventral tegmental area (VTA) to amygdala connectivity. Enjoying monetary gains was associated with VTA-to-NAc and amygdala-to-NAc connectivity. The mesolimbic dopamine system originates in the VTA and sends major projections to the amygdala and NAc. Specifically, sexual humor appreciation was associated with effective connectivity from the ventromedial prefrontal cortex (vmPFC) to the amygdala, suggesting that subjective pleasure triggers activation of the vmPFC which exerts an excitatory influence on the amygdala. Unexpectedly, processing pleasure from monetary gains was linked to VTA-to-vmPFC connectivity, rather than the expected vmPFC-to-NAc connectivity. Importantly, we identified core roles for the *amygdala*. Sexual humor appreciation was associated with VTA-to-amygdala and vmPFC-to-amygdala effective connectivity, while we found amygdala-to-lOFC connectivity for erotic pleasure and amygdala-to-NAc connectivity for pleasure from monetary gains. Our findings represent an important step in understanding how effective connectivity in the mesocorticolimbic-amygdala circuitry differs for processing the consumption of sexual and monetary rewards.

## 1. Introduction

An increasing number of studies have shown that the amygdala plays a key role in humor appreciation [1–14]. A large body of research has shown that the ventral striatum, especially the nucleus accumbens (NAc), is involved in the anticipation of monetary rewards [8, 9, 15–20]. The present study builds on our own earlier and more recent research on the reward consumption or outcome phase in the amygdala and midbrain for sexual humor rewards, the nucleus accumbens (NAc) for monetary rewards, and the lateral orbitofrontal cortex (lOFC) and hypothalamus for erotic rewards [9]. The results of previous studies have shown distinct patterns of functional connectivity (i.e., non-directional connectivity) in the *amygdala-midbrain* during both general humor and sexual humor appreciation [8, 9], *NAc-midbrain* during the experience of pleasure from monetary rewards [8], and *1OFC-amygdala* for sexual (erotic and sexual humor) rewards [9]. However, little is known about the patterns within the *effective connectivity* (i.e., directional connectivity) of the mesocorticolimbic (MCL) dopaminergic network during the consumption of sexual (erotic and sexual humor) and non-sexual (monetary) rewards.

In particular, the ventromedial prefrontal cortex (vmPFC) plays an important role in processing subjective value [19]. Recent neuroimaging studies have begun to identify the neural substrates of hedonic pleasure involved in encoding the subjective values of different types of rewards [21]. The vmPFC appears to play a critical role in computing expected value, reward outcome value and experienced pleasure for different stimuli, possibly playing a role in value-based decision-making processes [18, 19, 21]. Previous studies of humor found positive correlations between subjective funniness ratings and vmPFC activation levels, suggesting that higher levels of subjective liking are associated with higher activation levels in the vmPFC [22, 23]. Other studies of rewards have shown that vmPFC activity was positively correlated with measures of subjective liking for different reward types [24]. Several studies have indicated that the vmPFC is frequently modulated by social rewards and that its activity levels are positively correlated with individual differences in subjective value [25]. As of yet, little is known about how subjective value signals in the vmPFC that occur during reward consumption are modulated by information represented in effective connectivity patterns. In addition, whether the vmPFC serves as a source or destination in representing reward values within a subjective value-based pleasure process remains unclear. Importantly, in contrast to our earlier studies [8, 9], the present study used the vmPFC as a node and subjective hedonic ratings as covariates in the dynamic causal modeling (DCM) and parametric empirical Bayes (PEB) analyses. By including subjective ratings in our analysis, this study attempted to provide insights into the distinct effective connectivity patterns and connection strength between the vmPFC and mesocorticolimbic (MCL) networks during the consumption of sexual (erotic and sexual humor) and non-sexual (monetary) rewards with subjective hedonic ratings as covariates.

There is a large body of research on the neural processing of primary and secondary rewards [19], but less is known about differences in the processing of sexual rewards, especially for sexual humor rewards [9]. Humor, a form of social play, is a universal and fundamental human experience. It involves a series of psychological processes related to humor comprehension, humor appreciation, and laughter [5]. The pleasurable experience of amusement is elicited by the comprehension of humorous content. Sexual themes are one of the most prominent in humor [26–28]. While earlier studies have deepened our understanding of the neural correlates of humor appreciation in the amygdala [1–14, 29], less is known about the neural mechanisms and the precise types of effective connectivity underlying sexual humor appreciation [9]. Erotic stimuli depict content that directly triggers or fosters sexual arousal through mechanisms belonging to our ancient reward circuitry [30]. Such stimuli are ‘primary rewards’ that directly involve a biological drive and thus have an innate reward value [19, 30, 31]. Such stimuli are thus distinct from sexual humor, which is a type of ‘secondary reward’, gaining its value through learned associations and the inferences that emerge from them, through mechanisms associated with our higher-order social reward circuitry [9]. With hedonic ratings included, this study attempted to provide insight into the distinct effective connectivity patterns associated with the experiences of *sensory pleasure* in response to erotic stimuli and *higher-order social pleasure* during sexual humor appreciation [32]. In contrast to our previous research [9], this study attempted to provide insights into the distinct effective connectivity patterns between amygdala and lOFC during the consumption of erotic and sexual humor rewards with subjective hedonic ratings as covariates.

In this study, the use of sexual humor rewards also allows us to differentiate between effective connectivity patterns for the ‘liking’ of two distinct types of secondary rewards. Sexual humor rewards involve a process of humor comprehension requiring social inferencing and humor appreciation in the amygdala [9], whereas monetary rewards are direct cash incentives for the performance or attainment of tasks or goals in the NAc [8, 9]. Previous decision-making studies have shown that the NAc, vmPFC, OFC, and amygdala play a vital role in the construction of the ‘common neural currency’ [21, 33]. The vmPFC plays an important role in processing subjective value but little is known about how subjective value signals in the vmPFC that occur during the consumption of secondary rewards (sexual humor and monetary rewards) are modulated by information represented in effective connectivity patterns. By including subjective ratings in our analysis, this study attempted to provide insights into the distinct effective connectivity patterns associated with higher-order pleasure in the vmPFC: *material* pleasure for direct cash incentives and *social* pleasure for the appreciation of sexual humor that requires social inferencing for its understanding. Taken together, little is currently known about how the subjective value signals in the vmPFC for both high-order pleasures (sexual humor and monetary rewards) of secondary rewards are modulated by information represented in effective connectivity patterns. The main aim of this study was to use subjective value (by including subjective hedonic ratings as covariates in our analysis) to better understand the patterns between vmPFC to amygdala effective connectivity during the consumption of sexual humor rewards [22, 23] and vmPFC to NAc effective connectivity during the consumption of monetary rewards [19].

Hedonic consumption involves pleasure, and this pleasure is elicited by the mesocorticolimbic (MCL) circuitry [32]. The amygdala, NAc, lOFC, and vmPFC are all major dopaminergic targets that have been implicated in reward processes [19]. The NAc and OFC, both of which play critical roles in reward processing, are the main projection areas of two distinct MCL dopaminergic pathways, the mesolimbic and mesocortical pathways [19]. Previous studies have shown that the dopaminergic system that projects from the ventral tegmental area (VTA) of the midbrain to the NAc, amygdala, and to other forebrain sites, is the major substrate of pleasure and reward centers [34, 35]. Conversely, dopaminergic signals can interact with excitatory inputs carried by projections from the amygdala and prefrontal cortex (PFC) which are involved in emotion and memory consolidation for reward associations [36]. Convergent evidence suggests the important role of the MCL pathway in the pleasure experienced as a result of consuming rewards [19, 32, 37]. In contrast to our previous research [9], the present study added a new node in the vmPFC to include the subjective encoding of reward values. However, the underlying mechanisms determining how the cognition-emotion regions interact with each other in the MCL neurocircuitry (VTA, NAc, amygdala, lOFC, and vmPFC) and hypothalamus during the consumption of sexual and non-sexual rewards are still not clearly understood.

In classical dynamic causal modeling (DCM), a few models are specified, and these models differ in the presence or absence of the influence of experimental manipulations on certain connections [38]. However, the present study used a novel method of DCM with a parametric empirical Bayes (PEB) approach to investigate the effective connectivity of neural networks underlying an experimental effect [39, 40]. In contrast to classical DCM analyses involving model comparison [38], the key advantage of a DCM-PEB approach is that it removes the need to contend with the multiple-comparison problem [40–43].

Although previous studies have used classical DCM to analyze effective connectivity during the anticipation of monetary gains or losses [44, 45], relatively few studies have investigated the effective connectivity of the mesocorticolimbic (MCL) reward neurocircuitry during the consumption phase for different types of rewards (rather than a single reward type, such as monetary rewards and during the anticipation phase). To the best of our knowledge, no studies using DCM-PEB analysis have investigated effective connectivity to examine the consumption of sexual (erotic and sexual humor) and non-sexual (monetary) rewards, and particularly sexual humor rewards [9]. Therefore, in contrast to Bayesian model comparison (BMC) using a comparative hypothesis-driven model of the “specific connection types” via shrinkage priors (confirmatory PEB analysis), the present study conducted a more exploratory Bayesian model reduction (BMR) to automatically search over all reduced models (exploratory PEB analysis) to especially allow the six-node (VTA, NAc, amygdala, lOFC, vmPFC, and hypothalamus) complex model in the reward processing to be compared quickly and efficiently for a six-node model [42].

To contribute to a better understanding of this circuit of the MCL dopamine system *with* subjective hedonic ratings included, this study starts with four distinct aims, all related to the task of determining the nature of the effective connectivity network active during the consumption of sexual and monetary rewards using a novel DCM-PEB approach [39, 40], and especially sexual humor rewards. First, we expected the modulatory effects would show stronger activations (connection strength) and more distinct effective connectivity directional patterns among all three types compared to analyses without the ratings. Second, we expected to find the *amygdala* playing a key role for sexual humor amusement, the *NAc* playing a key role for the consumption of monetary gains, and the *lOFC* playing a critical role for erotic pleasure. In particular, we expected to identify effective connectivity between the amygdala and midbrain (VTA) for sexual humor appreciation [8, 9], while we expected modulatory changes of effective connectivity between the NAc and midbrain (VTA) for the pleasure experienced in receiving monetary gains [8]. We also expected to differentiate between patterns of effectivity connectivity involving the lOFC and amygdala associated with the hedonic enjoyment of different types of sexual rewards [9]. Third, and importantly, based on previous studies [19, 22, 23], we expected to identify patterns of effectivity connectivity between the vmPFC and amygdala for the hedonic pleasure associated with sexual humor rewards [22, 23], and effectivity connectivity between the vmPFC and NAc for the hedonic pleasure associated with monetary gains [19]. Finally, we expected to find the *amygdala* playing a core role [1–14, 29] and to identify distinct patterns of effectivity connectivity between the amygdala and mesocorticolimbic (MCL) networks for the consumption of sexual (erotic and sexual humor) and non-sexual (monetary) rewards.

## 2. Methods

### 2.1 Participants

The present study is based on a reanalysis of imaging data from a sample of thirty healthy male volunteers (mean age and SD = 24.37 ± 2.70) who participated in a previous study of Chan et al., 2022 [9]. Based on earlier research on the role of the hypothalamus in male sexual arousal [30, 46–48], we decided to use heterosexual males as participants in this study. All participants were right-handed (as assessed by Edinburgh Handedness Inventory) [49], had normal or corrected-to-normal vision and had no history of neurological or psychiatric problems. Written informed consent was obtained from each participant recruited for fMRI scanning under a protocol approved by the Research Ethics Committee of National Tsing Hua University in Taiwan.

### 2.2 Stimuli

To ensure that the erotic pictures and sexual humor cartoons were valid as stimuli, two behavioral studies were conducted. Both of these earlier studies are fully described in Chan et al., 2022 [9]. Sexual arousal was elicited through the physiological and psychological responses to sexual stimuli. The sexual humor stimuli used single-frame cartoons with an implicit or explicit reference to sexual content to elicit humor-related sexual arousal (high-order cognitive pleasure). The erotic stimuli depicted partially or completely naked women to directly induce sexual arousal (sensory pleasure). The sexual humor and erotic stimuli (i.e., sexual rewards) were designed based on relief, superiority, benign violation, and salience theories of humor [26, 28, 50–53]. The fMRI study used 32 erotic stimuli and 32 sexual humor stimuli selected through the two behavioral studies mentioned above [9], along with 32 monetary stimuli showing an image of coins and 32 non-reward stimuli showing gray scrambled images.

### 2.3 Experimental paradigm

The event-related fMRI experimental paradigm was also fully described in Chan et al. (2022) [9]. The ‘anticipation phase’ included four conditions: erotic anticipation (EA), sexual humor anticipation (HA), monetary anticipation (MA), and non-reward anticipation (NA). The ‘outcome phase’ included four corresponding conditions: erotic outcome (EO), sexual humor outcome (HO), monetary outcome (MO), and non-reward outcome (NO). The non-reward conditions (NA and NO) were baselines. The present study aimed to further distinguish between the patterns of neural effective connectivity associated with erotic stimuli, sexual humor, and monetary rewards during the anticipation and outcome phases with subjective hedonic ratings included.

All trials began with a fixation at the center of the screen with a jittered interval (mean = 500 ms). One of four visual reward cues (EA, HA, MA, and NA) appeared for 1000 ms, followed by an anticipatory delay period with a jittered interval of 2500 ms. Participants had 800 ms to respond to a numerical judgment task calling on them to indicate whether a target number was smaller than 5 (i.e., 1–4) using an index finger or larger than 5 (i.e., 6–9) using a middle finger on a right-hand button press. The number 5 was not shown. Next, participants were shown whether they had earned a reward (EO, HO, MO, and NO) for successful task completion on the trial (a ‘no reward’ outcome, indicated by a matched scrambled image, was earned in cases of participant errors or incomplete responses). This reward feedback phase lasted 7000 ms, allowing time for participants to comprehend the reward, particularly for the sexual humor condition. In trials where rewards were not given (i.e., non-reward condition), gray scrambled pictures were presented for both successful and unsuccessful trials. After the reward outcome display ended, participants were given 3000 ms to give a subjective hedonic rating expressing their level of enjoyment of the stimulus on a 1–4 continuous scale (1 = very little pleased; 4 = very highly pleased). Finally, a jittered intertrial interval (ITI; mean = 2500 ms) was presented (S1 Fig in S1 File).

There was a total of four runs, presented in counterbalanced order across participants. Each run consisted of 32 trials in random order, and included eight trials of each of the four experimental conditions: erotic, sexual humor, monetary, and non-rewards, for a total of 128 trials per participant. The average length across all trials was 17.3 s and the average length for each run was 9 min 20 s, with a 1-min break between runs. The total duration of the fMRI experiment was approximately 44 min per participant.

### 2.4 Image acquisition

Functional images were acquired using a 3T Siemens Magnetom Prisma scanner (Erlangen, Germany) using a standard 20-channel head coil at the Imaging Center for Integrated Body, Mind and Culture Research, National Taiwan University. Visual stimuli were presented using the software E-Prime 3 (Psychology Software Tools, INC) and through MRI-compatible goggles (Resonance Technology, Inc.) to enable participants to clearly see the details and lines for the sexual humor stimuli. Blood oxygenation level-dependent (BOLD)-sensitive T2*-weighted functional images were acquired using a single shot gradient echo-planar imaging (EPI) pulse sequence. Functional images parallel to the anterior-posterior commissure (AC-PC) and covering the whole brain were acquired per volume with the following parameters: repetition time (TR) = 2000 ms, echo time (TE) = 30 ms, flip angle (FA) = 90°, field of view (FOV) = 220 × 220 mm^2^, 64 × 64 matrix, voxel size = 3.43 × 3.43, 3.40 mm^3^, 36 interleaved slices, and 3.40 mm slice thickness with no gap. The first four volumes of each functional run were discarded to allow for T1 equilibration effect. Finally, each functional run consisted of 280 functional volumes. High-resolution structural T1-weighted anatomical images was acquired for each participant using a 3D gradient-echo pulse sequence with the parameters of TR = 1900 ms, TE = 2.28 ms, FA = 9°, FOV = 256 × 256 mm^2^, 256 × 256 matrix, voxel size = 1 × 1 × 1 mm^3^, and 192 slices.

### 2.5 Image analysis

A detailed description of the preprocessing, general linear model (GLM) analysis, and functional connectivity with psychophysiological interaction (PPI) analysis is provided in our previous study on the same dataset [9]. Briefly, we preprocessed and analyzed images using Statistical Parametric Mapping (SPM12) (Wellcome Department of Imaging Neuroscience, London, UK). The present study further used a DCM-PEB approach to measure effective connectivity for sexual (erotic and sexual humor) and non-sexual (monetary) rewards.

#### 2.5.1 Preprocessing

Preprocessing steps included slice time acquisition correction (referenced to midpoint of slice number), realignment of motion correction, spatial normalization into the Montreal Neurological Institute (MNI) template by means of the deformation field of coregistered, segmented structural data, and smoothing with an 8-mm full-width at half-maximum (FWHM) isotropic Gaussian Kernel to increase the signal-to-noise ratio (SNR).

#### 2.5.2 General linear model (GLM) analysis

Effects were estimated using the general linear model (GLM) with an event-related design as implemented in SPM12. At the first level (single-subject level), regressors were created for reward type (erotic, sexual humor, monetary, and non-reward) during the anticipation phase (EA, HA, MA, and NA) and the outcome phase (EO, HO, MO, and NO) with a canonical hemodynamic response function (HRF) with the delta functions modeled by event onset for each trial. Additionally, six motion parameters were entered as regressors of no interest.

At the second level (group level), region of interest (ROI) analysis was used. Based on our earlier study comparing monetary with humor rewards [8] and our results for the same dataset [9], the present study included the NAc as an ROI for monetary rewards and the amygdala and midbrain as ROIs for sexual humor rewards. Based on previous research comparing monetary with erotic rewards [30] and our brain activation results for the same dataset [9], the present study included the lOFC and hypothalamus as ROIs for erotic rewards. In sum, based on our earlier results with the same dataset [9], the present study included the NAc, amygdala, midbrain, lOFC, and hypothalamus as ROIs. Based on our previous studies [8, 9], the present study mainly focused on the ‘outcome phase’ because our interest was in the differences in effective connectivity during the hedonic enjoyment resulting from consumption of the sexual (versus non-sexual) rewards. Additionally, as we used in-scan subjective hedonic pleasure ratings in our analysis, we also included the vmPFC as an ROI. The vmPFC reflects the subjective value of feelings of amusement in response to humor [22, 23] and the pleasure arising from the receipt of monetary rewards [19, 32]. For these reasons, the vmPFC was also included as an ROI. Statistical significance was defined as family-wise error (FWE) corrected cluster probability *p* < 0.05 with a minimum of 10 voxels.

#### 2.5.3 Effective connectivity analysis: DCM analysis

The brain is a complex and dynamic functional system. Functional connectivity analysis seeks to measure non-directional correlations or co-activations between pairs of time series obtained from different brain regions (seed-ROI couplings) during task performance. By contrast, effective connectivity (EC) analysis seeks to identify directional relationships between brain regions (node → node) and the connection strengths (in Hz units) for each during the performance of some task [10, 54]. To investigate the consistency and specificity of reward-related pathways from functional connectivity studies [8, 9], the present study further used DCM analysis to identify the effective connectivity involved in the processing of sexual and monetary rewards.

We used DCM analysis to characterize the effective connectivity that explains BOLD responses to erotic, sexual humor, and monetary rewards during the anticipation and outcome phases. To quantify the effects of reward types (erotic, sexual humor, and monetary rewards) on effective connectivity between the nodes, we employed DCM to assess the causal information flow [41]. Further, we applied a PEB approach [40, 42] with DCM to obtain subject-specific estimates of these parameters after fitting group data. To further understand the patterns between effective connectivity for the types and the level of self-reported enjoyment, we modeled the hedonic ratings made by the participants during the outcome phase in the PEB analysis. The hedonic ratings for erotic, sexual humor and monetary rewards were mean-centered inputs and modeled as covariates.

DCM was implemented using the SPM12 to estimate effective connectivity (EC). DCM is a model describing directional neural activity by dynamic bilinear differential equations where the neural activity of a region and the time derivative can be determined by intrinsic connections (A matrix), experimental modulation connections (B matrix), and the directly experimental driving input (C matrix) [39] between the six spherical volumes of interest (VOIs; VTA, NAc, amygdala, lOFC, vmPFC, and hypothalamus) active during the processing of sexual (erotic and sexual humor) and non-sexual (monetary) rewards. These matrices all describe changes of connectivity in unit Hz. The diagonals of the A and B matrices stand for the values of self-inhibition connections, with values of -0.5 × exp(ɑ), where -0.5 Hz is the default value of the self-connection and unitless log-scaling parameter ɑ is estimated from the fMRI data [39]. A positive value of *a* indicates an increase in the inhibition of the region, representing reduced responsivity to the inputs from the network. A negative self-connection value indicates a decrease in the inhibition of the region. The other (non-diagonal) connections of the A and B matrices stand for the activity of an area (“destination”) that is caused by the change of activity in another area (“source”). Positive values stand for excitatory influences, while negative values represent inhibitory influences.

#### 2.5.4 Selection of DCM nodes

The candidates for a *priori* DCM nodes were determined in two steps. In step 1, based on our previous studies [9], five candidate nodes belonging to a reward circuit that showed significant activation (*p* < .05, FWE corrected) in the same dataset [9] were selected. These a *priori* selected candidate DCM nodes were as follows: the VTA of the midbrain, NAc, amygdala, lOFC, and hypothalamus [9]. In step 2, the present study mainly focused on the ‘outcome phase’. Several previous studies have found that the vmPFC covaried with subjective pleasure ratings and appears to play a key role in subjective reward value [19, 22–23, 25, 32]. A second-level conjunction analysis was conducted to identify brain areas that were activated in common for rewards [55]. Based on these criteria, the nodes of VTA of the midbrain, NAc, amygdala, lOFC, hypothalamus, and vmPFC were selected as the six DCM nodes. For each participant, the time series of each node, which was the BOLD signal over time in that node, was extracted by using the principal component (eigenvariate) of all voxels within each VOI of that node using the SPM12 eigenvariate toolbox. Each eigenvariate of the time series was also adjusted for the F-contrast modelling of the effects of interest (regressors) and then entered into the DCM analysis [56].

#### 2.5.5 Specification of the model: Driving inputs and modulation of connectivity

A fully connected six-node DCM (including self-connection of each node, for a total of 108 connections; 6 nodes × 6 nodes × 3 conditions) was specified and estimated. A bilinear DCM model was specified for each participant. The prior models for erotic, sexual humor and monetary rewards were specified as follows: (a) the driving input (all stimuli) was entered for all six nodes; (b) all the between-region endogenous connections were set to ‘on’ in the absence of experimental manipulations; and (c) the experimental manipulation effects (erotic, sexual humor, and monetary rewards) modulated all the endogenous connections. The “all reward”, “erotic reward”, “sexual humor reward”, and “monetary reward” conditions were entered as experimental conditions in each DCM model. All single-subject full models were collated in a MATLAB cell array (i.e., a group DCM or GCM). These full models were then estimated to find optimal input parameters and optimal modulation parameters to provide the highest model evidence in each participant.

#### 2.5.6 Parametric empirical Bayes (PEB) analysis of group effects

To estimate connectivity effects of group level parameters in the mesocorticolimbic (MCL) network of erotic, sexual humor, and monetary rewards for each participant, we conducted a second-level PEB analysis to ensure posterior densities over connectivity parameters (posterior means and covariances) for group effects [40, 42]. An advantage of PEB method, in contrast to classical random-effects (RFX) analysis [38], is that PEB not only takes the mean, but also considers the variability of individual connection strengths [40]. With the subjective hedonic pleasure ratings included, the group level analysis of each effective connections (a total of 108 tested connections) was performed using the PEB method. The PEB approach as implemented in SPM12 was used to conduct group-level analyses for effective connectivity modulatory changes, as follows: (a) testing the group effects for every DCM parameter and (b) estimating the model after including all the covariates. In this study, the covariate matrix of the PEB model included the following regressors: (i) the mean (ones for all subjects) and (ii) mean-centered hedonic pleasure ratings. The PEB model was then estimated by SPM 12 code (spm_dcm_peb.m).

To evaluate the distinct effective connectivity of three reward types with subjective ratings as covariates during the outcome phase, we then used Bayesian model reduction (BMR) [40, 42] to automatically search over PEB reduced models with group differences. BMR was employed to iteratively prune connection parameters which did not contribute model evidence for the rapid comparison of every reduced model underlying the six-node full PEB model. In contrast to a hypothesis-driven model using specific connection types to perform Bayesian model comparison (BMC) [40–42, 57, 58], we used a more exploratory BMR, performing an automated search over reduced PEB models (fully-connected and recurrent networks), which is a particularly efficient form of Bayesian model selection (BMS) [59], especially for a six-node complex full model. This search was accomplished with the simplifying assumption that these models were all equally likely a *priori*. The model evidence considers both model accuracy (how well the model fits the data) and model complexity (the difference between model parameters and their prior values). The BMR procedure was accomplished in an iterative process [42, 43].

Finally, we performed Bayesian model average (BMA) analysis to average the parameters across all models searched by the Bayesian model reduction (BMR). Estimating the parameters of all plausible DCM models can be a time-consuming process and novel ways have been developed to make BMA more computationally efficient [40]. Through BMR, a BMA was calculated over the 256 best models to obtain the optimized model effective connectivity parameters by weighting their posterior probability [43, 58]. In our present study, significant network parameters were determined with a posterior probability of Pp > 0.95, which represents the probability of the parameters being present versus absent.

## 3. Results

### 3.1 In-scan behavioral results

Participants rated the hedonic pleasure experienced in each condition (EO, HO, MO, and NO) on a 4-point scale (1 for very little pleased; 4 for very highly pleased) during the scanning procedure. The behavioral results during the scanning have been described in detail in Chan et al. (2022) [9]. Ratings for reward outcomes following successful trials were 3.42 ± 0.43 in the EO, 3.33 ± 0.32 in the HO, 3.67 ± 0.35 in the MO, and 1.75 ± 0.60 in the NO condition. A one-way repeated-measures ANOVA of these ratings was significant, *F*(3, 87) = 134.03, *p* < .001, η^2^ = .822. Bonferroni *post hoc* tests found that the ratings for monetary pleasure were highest, while ratings for both erotic pleasure and sexual humor amusement were higher than for non-rewards. A one-way repeated-measures ANOVA of the hedonic ratings for the non-reward image following unsuccessful trials showed no significant differences across four conditions, *F*(3, 21), = .59, *p* >.05, η^2^ = .078.

### 3.2 DCM results

The DCM results showed the intrinsic or endogenous connections (A matrix), modulatory effects (B matrix), and modulatory effects of participants’ hedonic ratings as covariates (B matrix) across the different reward types. To evaluate the modulatory effects of erotic, sexual humor and monetary rewards on effective connectivity during reward processing, we conducted a DCM analysis with a PEB approach. DCMs were specified separately for anticipation and outcome phases that included MCL networks. The diagonal of the matrix indicates the values of self-connections. A positive self-connection value indicates an increase in the inhibition of the region, while a negative self-connection value indicates a decrease in the inhibition of the region. The non-diagonal connections of the matrix stand for the activity from the source area to the destination area. Positive modulatory effects indicate that the source region exerts an excitatory influence on the activity of the destination or target region, while negative modulatory effects indicate that the source region exerts an inhibitory effect on the activity of a target region [40].

#### 3.2.1 Effectivity connectivity for erotic, sexual humor and monetary rewards during the anticipation phase

Monetary anticipation was associated with a significant positive effect of the VTA of the midbrain dopaminergic system on the rate of NAc activity. The strongest effect of monetary anticipation was an increase in excitatory coupling from the vmPFC to the NAc (vmPFC → NAc). Monetary anticipation was associated with excitatory coupling from the VTA to the NAc (VTA → NAc) and from the NAc to the lOFC. It was also associated with significantly negative coupling between the amygdala and the NAc, meaning that increased amygdala activity during monetary anticipation caused a decrease in NAc activity. Erotic wanting (i.e., anticipation) was associated with stronger connectivity from the VTA to the lOFC, while anticipation for sexual humor rewards was associated with stronger connectivity from the NAc to the hypothalamus and smaller modulatory changes in the hypothalamus to the vmPFC connection and in the hypothalamus to the VTA connection. Conversely, monetary cues had an influence on most connections throughout the NAc network. Effective connectivity during the monetary anticipation phase was shown in the S2 Fig in S1 File.

#### 3.2.2 Effectivity connectivity for erotic, sexual humor, and monetary rewards during the outcome phase—Without hedonic ratings

Effective connectivity during the ‘liking’ or outcome phase was first conducted without including the hedonic ratings as covariates. All EC values from the VTA to the other three nodes (hypothalamus, lOFC, and vmPFC) were for erotic rewards (VTA → hypothalamus; VTA → lOFC; VTA → vmPFC) in contrast to the values for the other two rewards (sexual humor and monetary gains). Notably, pleasure from erotic stimuli showed a key role for the vmPFC, which received excitatory influences from the VTA, lOFC, and hypothalamus (i.e., VTA → vmPFC; lOFC → vmPFC; hypothalamus → vmPFC). Also, erotic pleasure was associated with effective connectivity from the VTA to the lOFC, including not only forward connections (VTA → lOFC), but also inverse ones (VTA ← lOFC). The strongest effect for erotic pleasure was seen in the coupling of the hypothalamus to the vmPFC.

The effective connectivity network from the VTA to the hypothalamus was activated by each type of reward. The effective connectivity networks from the VTA to the lOFC (VTA → lOFC) and from the lOFC to the vmPFC (lOFC → vmPFC) were activated by sexual (erotic and sexual humor) rewards, but not by the pleasure of receiving monetary gains. Conversely, pleasure from receiving monetary gains activated a network from the lOFC to the VTA (lOFC → VTA) and from the VTA to the NAc (VTA → NAc). These results, analyzed without including participants’ hedonic ratings, provide initial insights into the processing of pleasure in response to rewards (S3 Fig in S1 File).

#### 3.2.3 Effectivity connectivity for erotic, sexual humor, and monetary rewards during the outcome phase—With hedonic ratings

**Fig 1:**
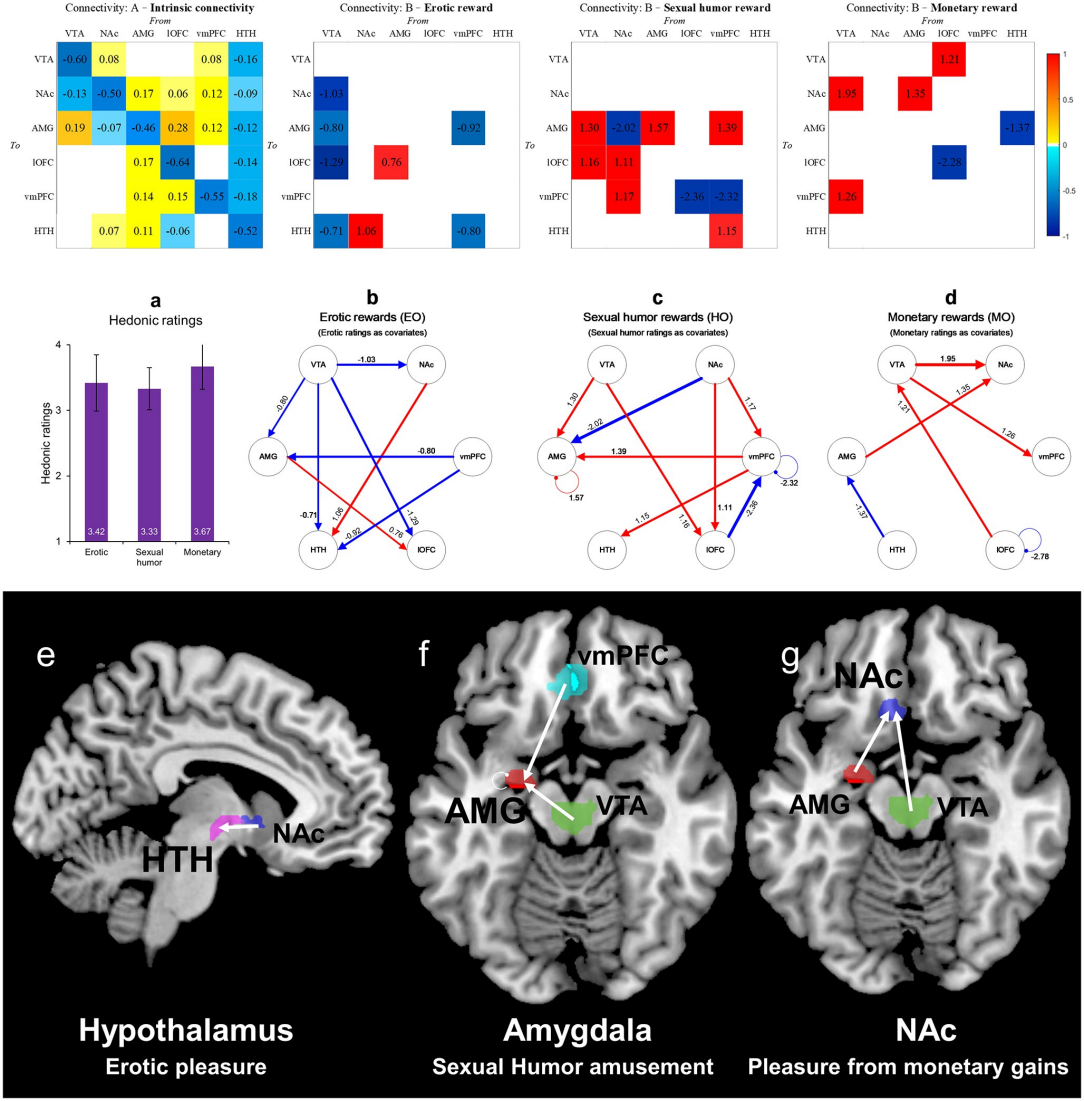
Effective connectivity in processing the consumption of erotic, sexual humor, and monetary rewards with hedonic ratings as covariates during the outcome phase. (Upper) Results of the DCM analysis are presented as connection weights given by the time constants in Hz for intrinsic connections (A matrix) and bilinear moderators (B matrix) for which significant effects (at 99% confidence). (Middle) The red lines represent positive modulatory effects and blue lines represent negative modulatory effects. Self-connections are displayed in the figure. (a) Hedonic ratings of erotic, sexual humor and monetary rewards from in-scan behavioral ratings on a 4-point Likert scale. Error bars represent the standard deviation (SD) of the mean. (b) Modulatory effect of pleasure from erotic images with ratings as covariates. (c) Modulatory effect of amusement from sexual humor with ratings as covariates. (d) Modulatory effect of pleasure from monetary rewards with ratings as covariates. (Lower) The hedonic ratings from in-scan data as covariates for erotic, sexual humor, and monetary rewards are associated with the hypothalamus, amygdala, and NAc, respectively. (e) Erotic pleasure was associated with a connection from the NAc to the hypothalamus. (f) The experience of amusement from sexual humor was associated with a connection from the VTA to the amygdala and from the vmPFC to the amygdala. (g) The experience of pleasure from monetary gains was associated with connections from the VTA to the NAc and from the amygdala to the NAc.

Effective connectivity (EC) analysis for the ‘liking’ or outcome phase was then conducted *with* the hedonic ratings (i.e., in-scan rating) included as covariates. With the hedonic ratings included, the modulatory effect indicated stronger activations (connection strength) and more distinct EC patterns for all three reward types (Fig 1) compared to analyses without the ratings (S3 Fig in S1 File). Interestingly, with the ratings included, the modulatory changes in the lOFC and hypothalamus for erotic pleasure, in the amygdala for sexual humor amusement, and in the NAc for pleasure from monetary gains showed EC patterns. With the hedonic ratings, self-connections are displayed. Positive numbers for the self-connections demonstrate increased self-inhibition and negative numbers for the self-connections indicate disinhibition.

**Fig 2:**
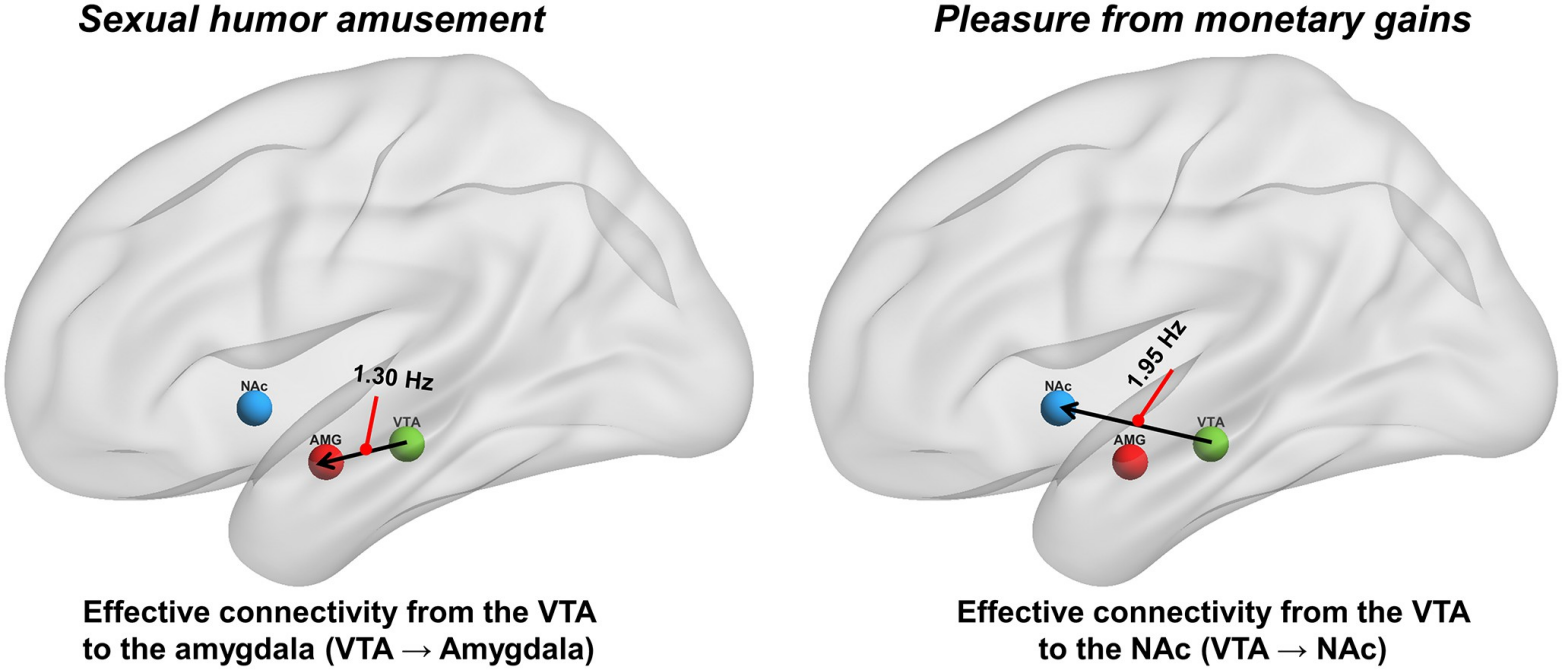
Effective connectivity in processing the consumption of sexual humor and monetary rewards with hedonic ratings as covariates during the outcome phase in the VTA, amygdala, and NAc. (Left) Sexual humor appreciation was associated with modulatory changes (connection strength = 1.30 Hz) in the effectivity connectivity from the VTA to the amygdala (VTA → amygdala). (Right) Experiencing pleasure from monetary gains was associated with modulatory changes (connection strength = 1.95 Hz) in the effectivity connectivity from the VTA to the NAc (VTA → NAc).

Notably, the processing of erotic rewards involved more distinct coupling patterns between the hypothalamus and the lOFC (Fig 1) than were found in analyses that did not include the ratings as covariates (S3 Fig in S1 File). Further, erotic rewards were associated with stronger connection strength but only two distinct positive modulatory EC change patterns in the ‘with ratings’ analysis (Fig 1), fewer than in the ‘without ratings’ analysis (seven positive modulatory changes) (S3 Fig in S1 File). Analysis of erotic pleasure with ratings as covariates found a stronger NAc to hypothalamus connection and a smaller amygdala to lOFC (amygdala → lOFC) connection (Fig 2). With the ratings as covariates, we did not find any significant positive modulation of the connections from the VTA during the experience of erotic pleasure. With the ratings as covariates, the results did not show EC from the VTA to the hypothalamus nor from the VTA to the lOFC.

Conversely, for sexual humor and monetary rewards, the analysis with ratings found increased positive EC and stronger connection strength. Analysis of the results for amusement from sexual humor with hedonic ratings as covariates showed modulation of the strength of the amygdala connections (Fig 1). In the analysis without ratings as covariates, there was no significant influence on the amygdala (S3 Fig in S1 File). The modulatory effects for sexual humor rewards with the ratings included involved stronger connections from the VTA to the amygdala (VTA → amygdala) and from the vmPFC to the amygdala (vmPFC → amygdala). Additionally, the modulatory effect of pleasure from sexual humor showed that NAc activity causes a decrease in the rate of amygdala activity (NAc ⇢ amygdala). The amygdala had a positive self-connection, suggesting an increase in the inhibition of the amygdala during sexual humor amusement.

Analysis of the results of pleasure from monetary gains with hedonic ratings as covariates showed greater modulatory changes in the NAc than was the case with sexual (erotic and sexual humor) rewards. VTA to NAc (VTA → NAc) and amygdala to NAc (amygdala → NAc) effective connectivity showed modulatory changes for the processing of pleasure for monetary gains (Fig 1).

Our previous studies using psychophysiological interaction (PPI) analysis identified functional connectivity in the amygdala-midbrain coupling during general humor appreciaiton [8] and sexual humor appreciation [9] during the outcome phase. The present study using DCM-PEB analyses with hedonic ratings found effective (directional) connectivity for sexual humor amusement from the VTA of the midbrain to the amygdala (VTA → amygdala) (Fig 2).

Previous monetary reward studies have found a key role for the NAc during the anticipation phase [15, 16]. Our previous research using PPI analysis further showed functional connectivity in the NAc-midbrain coupling during the outcome phases [8]. The present study using DCM with a PEB approach with hedonic ratings as covariates found effectivity connectivity for monetary rewards from the VTA of the midbrain to the NAc (VTA → NAc) during the outcome phase (Fig 2).

Previous research on reward value coding during the consumption of erotic and monetary rewards has highlighted, the key role of the lateral orbitofrontal cortex (lOFC) [19, 30]. One of our recent studies found evidence for a key role for the lOFC in distinguishing between sexual (erotic and sexual humor) and non-sexual (monetary) rewards [9]. The present study using DCM with PEB analyses of fMRI data with hedonic ratings as covariates further identified distinct effectivity connectivity patterns.

**Fig 3:**
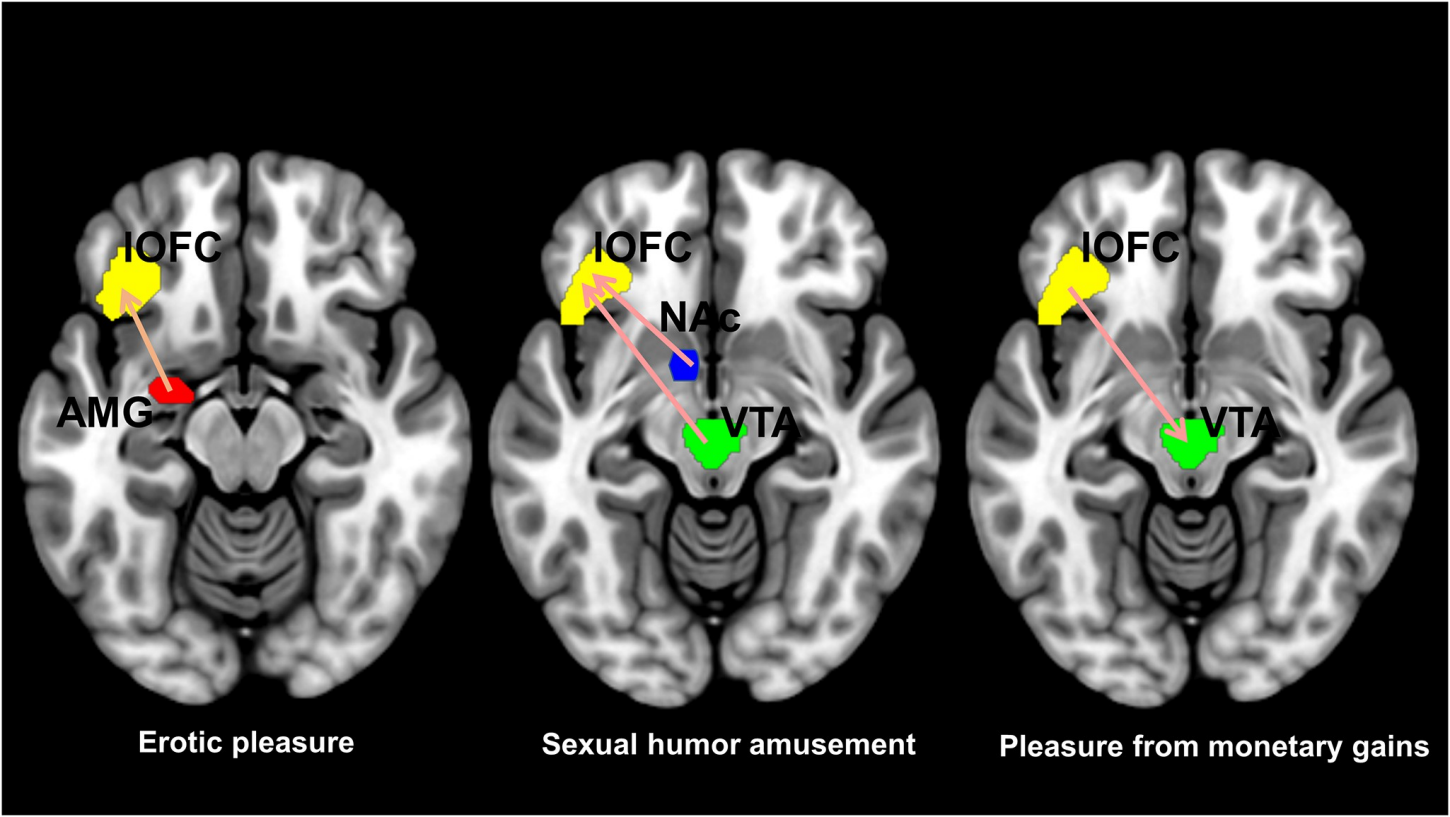
Effective connectivity in processing the consumption of sexual rewards and non-sexual (monetary) rewards with hedonic ratings as covariates during the outcome phase in the lateral orbitofrontal cortex (lOFC). (Left) During the processing of erotic pleasure, there were modulatory changes in amygdala to lOFC (amygdala → lOFC) effectivity connectivity. (Middle) Sexual humor appreciation was associated with stronger modulatory changes in VTA to lOFC (VTA → lOFC) and NAc to lOFC (NAc → lOFC) effectivity connectivity. (Right) Experiencing pleasure from monetary gains was associated with stronger modulatory changes in lOFC to VTA (lOFC → VTA) effectivity connectivity.

Sexual (erotic and sexual humor) rewards with hedonic ratings as covariates were associated with greater modulatory changes in the lOFC than were monetary rewards during the outcome phase. Amygdala to lOFC (amygdala → lOFC) effectivity connectivity showed modulatory changes in response to erotic pleasure. Also, VTA to lOFC (VTA → lOFC) and NAc to lOFC (NAc → lOFC) effectivity connectivity showed modulatory changes in response to sexual humor appreciation (Fig 2). However, lOFC to VTA (lOFC → VTA) EC showed modulatory changes in response to the pleasure from experiencing monetary gains. The lOFC had a negative self-connection in response to monetary rewards, suggesting a decrease in the inhibition of the lOFC during consumption of monetary gains (Fig 3).

Previous studies of humor appreciation with subjective funniness ratings as covariates have indicated a critical role for the vmPFC [22, 23] and studies with monetary rewards have found a role for the vmPFC in processing subjective value [19, 32]. The present study used the vmPFC as a node to further investigate the patterns of effective connectivity associated with different reward types, with a focus on the processing of subjective value. Monetary and sexual humor rewards with hedonic ratings as covariates were associated with greater modulatory changes in the vmPFC than were erotic rewards. Also, vmPFC → amygdala EC was found for sexual humor appreciation, while VTA → vmPFC EC was found for the processing of pleasure from monetary gains.

**Fig 4:**
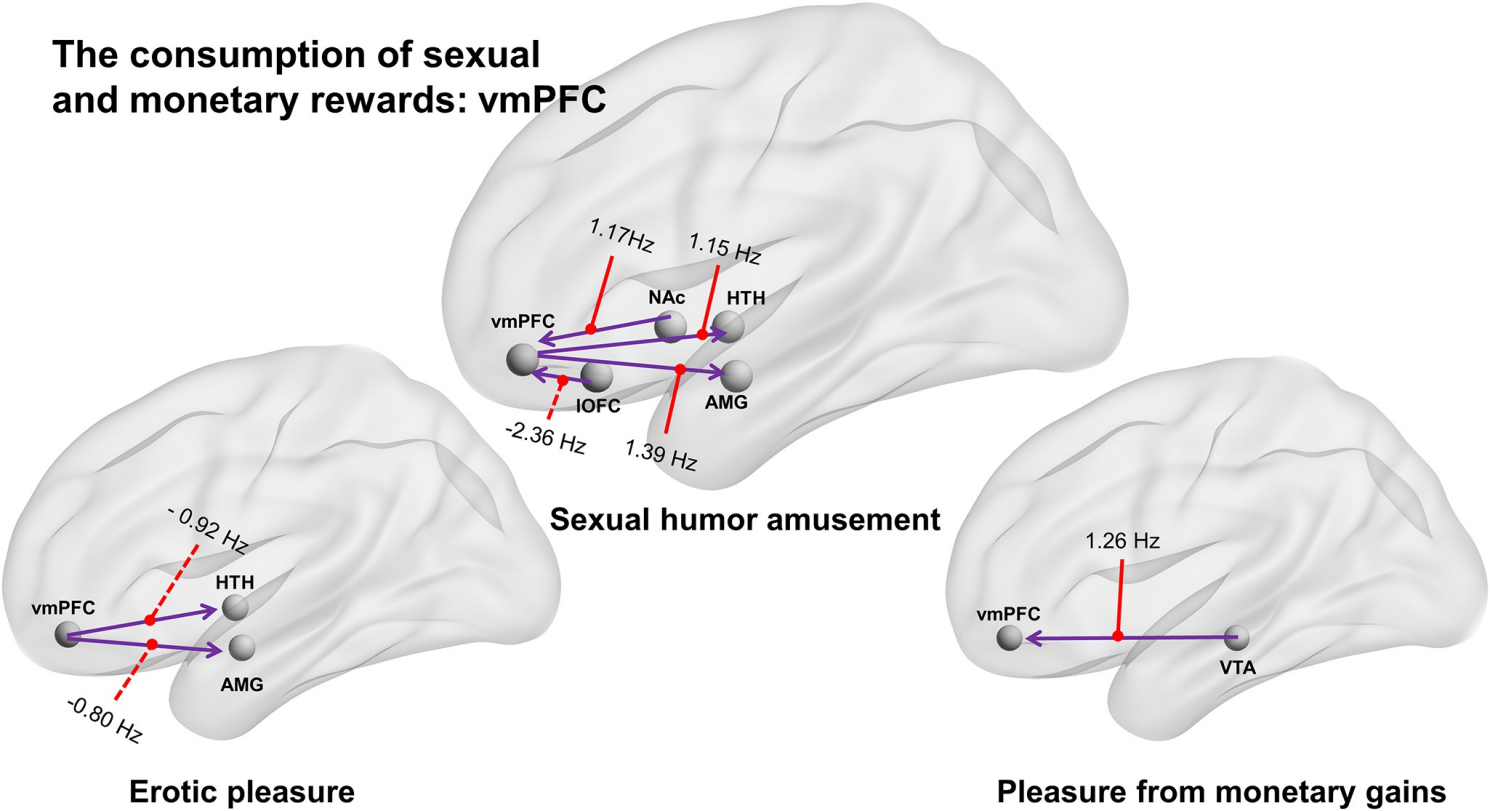
Effective connectivity in processing the consumption of erotic, sexual humor and monetary rewards with hedonic ratings as covariates during the outcome phase in the vmPFC. (Left) Erotic pleasure was associated with negative modulatory effects from the vmPFC to both the amygdala and hypothalamus (vmPFC ⇢ amygdala; vmPFC ⇢ hypothalamus). (Middle) Conversely, sexual humor appreciation was associated with positive modulatory effects in the effectivity connectivity from the vmPFC to the amygdala (connection strength = 1.39 Hz). Also, sexual humor appreciation was associated with positive modulatory changes in effectivity connectivity from the vmPFC to the hypothalamus (vmPFC → hypothalamus) and from the NAc to the vmPFC (NAc → vmPFC). (Right) Pleasure from monetary gains was associated with positive modulatory changes of effectivity connectivity from the VTA to the vmPFC (VTA → vmPFC; connection strength = 1.26 Hz).

In addition, NAc to vmPFC (NAc → vmPFC) and vmPFC to amygdala and hypothalamus (vmPFC → amygdala; vmPFC → hypothalamus) effectivity connectivity showed modulatory changes in response to sexual humor rewards. The positive modulatory effects of amusement from sexual humor resulted in stronger connections from the vmPFC to both the amygdala and hypothalamus (connection strength = 1.39 Hz and 1.15 Hz), while the negative modulatory effects of pleasure from erotic stimuli resulted in stronger connections from the vmPFC to both the amygdala and hypothalamus (connection strength = -0.80 Hz and -0.92 Hz) (Fig 4).

## 4. Discussion

While there is a large body of research on the neural mechanisms of monetary rewards [8–9, 15–20, 30, 60], less is known about the effective connectivity involved in the appreciation of sexual rewards [9]. In this study, we used DCM with a PEB approach to attempt to identify the patterns of the information flow within the reward network comprising a set of six nodes, including the VTA of the midbrain, NAc, amygdala, lOFC, vmPFC, and hypothalamus during the anticipation and consumption of erotic, sexual humor, and monetary rewards. This study segregated the effective connectivity (EC) of ‘liking’ (implicit and subjective hedonic experiences) for erotic, sexual humor and monetary rewards during the outcome or consumption phase. Our analytical focus was on the consumption of these rewards using subjective hedonic ratings as covariates, rather than on the results without the ratings as covariates. With the hedonic ratings included, the modulatory effects indicated stronger activations (connection strength) and more distinct EC patterns among all three types (Fig 1) compared to analyses of the consumption phase without the ratings (S3 Fig in S1 File), especially in sexual humor reward. This is relevant to the first aim of the present study. Given the lack of a well-developed research literature and the absence of strong hypotheses concerning “neural models” for responses to sexual and non-sexual rewards (especially sexual humor rewards), we chose to use an exploratory automatic search analysis (Bayesian model reduction, BMR) instead of a family-level model comparison method (Bayesian model comparisons, BMC) [59]. Future studies might further investigate the EC related to processing the consumption of sexual and non-sexual reward circuits with and without subjective hedonic ratings as covariates to evaluate how the connectivity in the ‘with ratings’ condition differs from that in the ‘without ratings’ condition using BMC based on the result of the current study.

Using hedonic ratings as covariates in our analysis, we identified core roles for the *amygdala* in the consumption of sexual humor, the *NAc* for the consumption of monetary gains, and the *lOFC* for the consumption of erotic stimuli. This is relevant to the second aim of the present study. Our findings are consistent with the existence of a mesocorticolimbic (MCL) center for rewards [18, 32, 61, 62] and represent an important step toward providing a directed coupling (effective connectivity) explanation for both sexual (erotic and sexual humor) and non-sexual (monetary) rewards. This is consistent with earlier findings that the amygdala plays a core role in the hedonic brain during the appreciation of general humor rewards [1–8, 10–14, 63–67] and sexual humor rewards [9]. This is also consistent with previous findings that the lOFC contribute to erotic pleasure [9, 19, 30]. A large body of previous studies have suggested that the NAc serves as a monetary incentive center during the anticipation phase [e.g., 8, 9, 15, 18]. This study also provides additional insights into the role of the NAc in the experience of pleasure from monetary gains during the outcome or consumption phase.

Furthermore, the second aim of the current study was to use effective connectivity analysis with a DCM-PEB approach to identify patterns between the amygdala and midbrain (VTA) during sexual humor appreciation [8, 9], between the NAc and midbrain (VTA) during the processing of pleasure from monetary gains [8], and between the amygdala and lOFC during the processing of pleasure from sexual rewards [9]. With subjective hedonic ratings included, the results of the present study confirmed the importance of increased EC from the VTA to the amygdala (VTA → amygdala) during sexual humor appreciation, increased EC from the VTA to the NAc (VTA → NAc) during the enjoyment of monetary gains, and increased EC from the amygdala to the lOFC (amygdala → lOFC) during erotic pleasure (Fig 1). These findings suggest distinct patterns of effective connectivity associated with sexual and non-sexual rewards, with mesolimbic and mesocortical dopaminergic pathways, especially in the roles of amygdala (sexual humor rewards), NAc (monetary rewards), and lOFC (erotic rewards).

The mesolimbic system contains dopamine neurons originating near or in the VTA of the midbrain, which chiefly ascend to the ventral striatum (especially in NAc) and to the amygdala [32]. The source of dopaminergic input, the VTA, is a main source of dopamine in the brain and has mostly been considered as a reward structure, which establishes and maintains reinforcement [68]. The VTA plays an important function in the mesolimbic dopamine pathway in modulating the extent to which limbic structures can exert an influence on humor appreciation and sexual arousal through the amygdala. The VTA may serve as a direct pathway and play a critical role in providing in modulating amygdala responses (VTA → amygdala; connection strength = 1.30) during sexual humor appreciation (Fig 2).

The present study also revealed VTA to NAc (VTA → NAc) EC during both monetary motivation (connection strength = 0.36) and the consumption of monetary rewards with (connection strength = 1.95) and without (connection strength = 0.35) subjective hedonic ratings, suggesting a ‘common neural currency’ circuit [32]. As suggested by previous studies, the NAc and VTA are thought to play key roles in representing reward value, especially for monetary rewards [8]. The present study further provides evidence of VTA → NAc effective (directional) connectivity in response to (a) anticipatory wanting in detecting the cued stimuli and (b) pleasure from monetary gains (with and without hedonic ratings as covariates). Taken together, the study found that the processing of sexual humor rewards involves a VTA → amygdala pathway, while processing monetary rewards involves a VTA → NAc pathway (Fig 2).

A large body of previous studies have suggested that the OFC links reward to hedonic experience [21, 69]. Interestingly, the second aim of the current study was to use a DCM-PEB approach to effective connectivity analysis to investigate connectivity between the lOFC and the amygdala during the consumption of sexual (erotic and sexual humor) rewards [9]. In the study, the lOFC was used as a reward role to make it possible to distinguish between sexual and non-sexual rewards [9, 19, 30, 31]. This is consistent with a view in which the lOFC plays a key role in the hedonic brain during the experience of pleasure in the consumption of erotic stimuli and sexual humor [9]. However, our own previous study found increased lOFC-amygdala functional connectivity in response to sexual rewards, including both erotic and sexual humor rewards [9]. The present study showed amygdala → lOFC effective connectivity only in the sensory pleasure during erotic rewards, not in high-order social pleasure during sexual humor appreciation (Fig 3). In other words, erotic pleasure was associated with amygdala to lOFC EC, while sexual humor appreciation was associated with VTA-to-lOFC and NAc-to-lOFC ECs, suggesting a mesolimbic-lOFC network underlying sensory and higher-order social pleasure for encoding the appreciation of sexual rewards. Although this was consistent with EC in the NAc → lOFC during sexual humor appreciation in our previous study [9], sexual humor appreciation was not associated with modulatory changes in amygdala → lOFC effective connectivity in this study. These results showed dopamine projections (VTA and NAc) to the prefrontal cortical region (lOFC) for sexual humor appreciation, while erotic pleasure was associated with amygdala → lOFC effective connectivity.

In particular, when contrasting effective connectivity during the consumption of primary (erotic) and secondary (sexual humor and monetary) rewards using subjective hedonic ratings as covariates, we also found that subjective pleasure involves VTA projections for secondary rewards (sexual humor and monetary rewards) but not primary erotic rewards (Fig 1). The VTA of the mesolimbic pathway plays a role in motivation and the subjective perception of pleasure [32]. However, without subjective ratings included, the VTA plays an important role in the VTA to lOFC and VTA to hypothalamus effective connectivity for erotic pleasure (S3 Fig in S1 File). Moreover, our analyses without subjective ratings included found a pattern of increased bidirectional effective connectivity between the VTA and the lOFC for erotic pleasure (S3 Fig in S1 File). However, subjective experiences of erotic pleasure may not necessarily be a completely accurate guide to the underlying neural mechanisms. Future studies might further investigate the EC related to processing erotic pleasure in the VTA, lOFC, amygdala, and NAc circuits with and without subjective hedonic ratings as covariates using Bayesian model comparison (BMC).

Importantly, the third aim of the current study was to use a DCM-PEB approach to examine vmPFC and amygdala effective connectivity for sexual humor appreciation and to examine vmPFC and NAc effective connectivity for the pleasure from receiving monetary gains, using subjective hedonic ratings as covariates (Fig 4). The vmPFC has been shown to be active in the processing of subjective value during humor appreciation [22, 23]. Our findings, when hedonic ratings were included as covariates, are consistent with earlier findings that the vmPFC and amygdala are active during humor appreciation [1, 2, 29]. The present study further found modulatory changes to vmPFC → amygdala effective connectivity (connection strength = 1.39 Hz) in the *high-order social pleasure* during sexual humor amusement (Fig 4), suggesting that subjective pleasure triggers activation of the vmPFC and then excitatory influence of the amygdala. These findings suggest that EC in the mesolimbic pathway during the appreciation of sexual humor rewards can, to some extent, be predicted by subjective hedonic pleasure. The greater the level of activation in the vmPFC (subjective outcome value), the higher the level of sexual humor appreciation in the amygdala.

Earlier studies of decision-making have shown that the NAc and vmPFC play a key role in the common neural currency [24, 32, 70]. Most earlier studies of anticipation have involved varying the choices, probabilities or values of anticipated monetary rewards [30] to explore neural responses. The present study used a range of 10 to 12 New Taiwan dollars (about USD 0.35 to 0.42) per trial to operationalize decision-making under uncertainty. We found that during the processing of *high-order material pleasure* from receiving monetary gains, there was a significant modulatory effect in VTA to vmPFC (VTA → vmPFC; connection strength = 1.26 Hz) effective connectivity (Fig 4). Unexpectedly, we did not find vmPFC to NAc (vmPFC → NAc) EC during the processing of receiving monetary rewards. However, monetary anticipation demonstrated highly significant modulatory effects in vmPFC to NAc (vmPFC → NAc; connection strength = 0.79 Hz) effective connectivity (S2 Fig in S1 File). Future studies might further examine the modulatory messaging from the vmPFC to the NAc for monetary motivation and from the VTA to the vmPFC for processing receiving monetary rewards for different tasks varying in intensity.

With the hedonic ratings included, *erotic pleasure* seemed to involve amygdala to lOFC (amygdala → lOFC) effective connectivity, while *erotic arousal* seemed to involve NAc to hypothalamus (NAc → hypothalamus) connectivity. In contrast with sexual humor appreciation and the arousal resulting from sexual humor, erotic pleasure and erotic arousal seem *not* to be elicited by subjective value in the vmPFC. Our results provide insight into understanding the relationship between sexual pleasure and sexual arousal with subjective encoding value in the amygdala → lOFC EC for erotic pleasure and NAc → hypothalamus EC for erotic arousal.

The particular features of sexual humor have been the focus of many humor theories. Relief theories have conceptualized the amusement experienced from sexual humor as related to a relief of tension arising from either physiological arousal or from the existence of ‘forbidden’ sexual and aggressive impulses [50]. Freud’s psychoanalytic theory suggested that the pleasure derived from such humor results from the saving of psychological energy usually spent on the inhibition of repressed impulses [50]. In short, people enjoy sexual humor because it allows them to briefly experience the illicit pleasure of releasing latent primitive sexual impulses. Previous studies have indeed found the enjoyment of sexual humor to be associated with repression and release of libido drives [26, 28]. Hobbes’ superiority theory proposes that the amusement we experience in response to humor arises from a sense of sudden triumph or feeling of superiority over another person or group [71, 72]. Benign violation theory proposes that humor arises from the perception of a violation (i.e., incongruity) in social norms that is interpreted as benign or harmless, and thus humorous [52, 53]. Also, salience theory has focused on content salience (e.g., sex and irony), providing a theoretical alternative to Freudian approaches [26, 51]. Psychoanalytic (relief), superiority, benign violation and salience theories, thus, have all attempted to explain the distinct characteristics of humor involving sexual content. These theories all suggest that the relief resulting from a release of tension contributes to the enjoyment people experience from sexual humor.

The MCL system (VTA, NAc, amygdala, lOFC, and vmPFC) is major dopaminergic sources or targets that have been implicated in reward processes [18, 19, 32]. Previous studies of humor have found activity in the vmPFC to be correlated with subjective pleasure [22, 23]. The present study found that the amygdala received convergent excitatory inputs from both the VTA and the vmPFC during the consumption of sexual humor rewards. The amygdala receives projections from a number of cortical regions [19, 73], but is most strongly connected to the OFC and NAc [18]. However, in this study, the amygdala received projections from the vmPFC and VTA. According to salience theory, sexual humor involves attention to sexually salient stimuli [26, 28, 51]. Sexual humor appreciation of social pleasure appears to primarily involve the midbrain-amygdala connection [9]. In this study, at the neurological level, the *amusement generated by sexual humor* seems to involve NAc to vmPFC and then vmPFC to amygdala (NAc → vmPFC → amygdala) effective connectivity, while *sexual humor arousal* seems to involve NAc to vmPFC and then vmPFC to hypothalamus (NAc → vmPFC → hypothalamus) connections (Fig 1).

Our previous research using a non-sexual humor (general humor) condition showed functional connectivity in the amygdala-midbrain coupling [8]. The present study using a sexual humor condition showed effective connectivity between the midbrain (VTA) and amygdala. The amygdala is involved in processing non-sexual humor and sexual humor [8, 9]. However, the present study additionally used subjective hedonic ratings of sexual humor as covariates. This study further showed effective connectivity between the vmPFC and the amygdala. The results might show humor appreciation processing in VTA to amygdala pathways, and especially the subjective appreciation of sexual content in vmPFC to amygdala connections. Future studies might combine the sexual (erotic and sexual humor) rewards and compare them with non-sexual humor (general humor) rewards and non-sexual (monetary) rewards.

Importantly, the present study showed that the amygdala receives multiple excitatory sources from both the VTA and the vmPFC (VTA → amygdala, vmPFC → amygdala) during the consumption of sexual humor rewards. However, in terms of modulatory effects during responses to sexual humor, NAc activity caused a decrease in the rate of amygdala activity (NAc ⇢ amygdala) during sexual humor appreciation (Fig 1). Future studies might further examine the negative modulatory messaging from the NAc to the amygdala for different types of humorous rewards. During the appreciation of sexual humor analyzed with ratings as covariates, the amygdala had a positive self-connection, suggesting an increase in the self-inhibition of the amygdala, while the vmPFC had a negative self-connection, representing a decrease in the self-inhibition of the vmPFC. The greater the positive self-connection, the more self-inhibited the region, suggesting that it was less sensitive to inputs from other nodes in the network [39, 40]. Increased EC from the VTA and vmPFC to the amygdala was found during sexual humor appreciation, suggesting that perhaps this circuitry is involved in amusement experiences with an increase or decrease in self-inhibition during sexual humor appreciation.

Finally, the fourth aim of the current study was to use a DCM-PEB approach to investigate the core role of the amygdala within the MCL system for sexual and non-sexual (monetary) rewards. With hedonic ratings included, sexual humor appreciation was associated with VTA to amygdala (VTA → amygdala) and vmPFC to amygdala (vmPFC → amygdala) effective connectivity (EC), while erotic pleasure was associated with amygdala to lOFC (amygdala → lOFC) EC and the pleasure from receiving monetary gains was associated with amygdala to NAc (amygdala → NAc) EC. For sexual rewards, the amygdala served primarily as a target, while for erotic and monetary rewards, the amygdala was a source. Our results provide a better understanding of the amygdala and MCL neurocircuitry (*MCL-amygdala*) active during the consumption of erotic, sexual humor, and monetary rewards. In sum, our results provided our understanding of the *MCL-amygdala* circuitry active during the consumption of erotic (amygdala → lOFC), sexual humor (vmPFC → amygdala and VTA → amygdala), and monetary rewards (amygdala → NAc), especially for sexual rewards in the *prefrontal-amygdala* circuitry.

## 5. Conclusions

This study of the processing of erotic, sexual humor, and monetary rewards provided evidence supporting the importance of reward effective connectivity involving six brain regions: the VTA of the midbrain, NAc, amygdala, lOFC, vmPFC, and hypothalamus. In this study, we mainly focused on the ‘outcome phase’ in the mesocorticolimbic (MCL) reward network because we were interested in differences in effective connectivity related to the hedonic enjoyment of consumption for our three reward types with subjective hedonic ratings as covariates. Building on our previous studies [8, 9], the present study further used a DCM-PEB approach to investigate the existence of connectivity between the amygdala and midbrain during sexual humor appreciation [8, 9], the existence of connectivity between the NAc and midbrain during the processing of pleasure from monetary gains [8], and the existence of connectivity between the lOFC and amygdala during sexual pleasure [9].

With the subjective hedonic ratings included, the amygdala, NAc, and lOFC were found to be major dopaminergic targets for sexual (erotic and sexual humor) and non-sexual (monetary) rewards. Findings on modulatory effects indicated stronger activations (connection strength) among all three types and a greater number of distinct EC patterns in the sexual humor rewards (Fig 1) compared to analyses without the ratings (S3 Fig in S1 File). We identified core regions for the processing of different reward types during the outcome phase with subjective hedonic ratings as covariates, including the *amygdala* (VTA → amygdala) for sexual humor appreciation, *NAc* (VTA → NAc) for monetary pleasure, and *lOFC* (amygdala → lOFC) for erotic pleasure. We also found that subjective pleasure involves VTA projections for secondary rewards (sexual humor amusement and monetary pleasure) but not primary rewards (erotic pleasure).

Consistent with previous studies on humor processing [22, 23], monetary rewards [15–19], and decision-making [24, 32, 70], subjective hedonic liking was correlated with monetary anticipation and gains and humor appreciation in the vmPFC. The present study showed distinct effective connectivity patterns for sexual and non-sexual rewards in the vmPFC. Sexual humor appreciation was associated with vmPFC → amygdala EC. However, monetary pleasure was associated with modulatory changes in VTA to vmPFC (VTA → vmPFC) EC during the outcome phase. The study also found modulatory changes in vmPFC to NAc (vmPFC → NAc) EC during the anticipation of monetary rewards. However, the present study did not find modulatory changes in NAc to vmPFC EC during the processing of pleasure from monetary gains during the outcome phase. In sum, monetary anticipation demonstrated vmPFC to NAc EC. Unexpectedly, monetary pleasure exhibited VTA to vmPFC EC, not vmPFC → NAc EC.

Importantly, we identified core roles for the amygdala in the consumption of sexual and non-sexual rewards. The study found modulatory changes in the *amygdala* (VTA → amygdala and vmPFC → amygdala) for sexual humor appreciation, *NAc* (amygdala → NAc) for the pleasure associated with monetary gains, and *lOFC* (amygdala → lOFC) for erotic pleasure.

In sum, the present study of hedonic pleasure processing found that sexual humor appreciation was associated with VTA → amygdala and vmPFC → amygdala effective connectivity. The processing of erotic pleasure was associated with amygdala → lOFC and NAc → hypothalamus effective connectivity. The processing of pleasure from monetary rewards was associated with VTA → NAc and amygdala → NAc effective connectivity. Unexpectedly, the processing of pleasure from monetary gains was also associated with VTA → vmPFC connectivity, rather than the expected vmPFC → NAc connectivity. Future studies might extend the circuit of interest to understand personality-based differences (e.g., high and low sense of humor) in the processing of sexual versus non-sexual rewards using Bayesian model comparison (BMC) in the MCL-amygdala neurocircuitry based on the result of the current study.

## Supporting information

S1 File (DOCX)
