# Supplementary material for: Effective connectivity of the amygdala during the consumption of erotic, sexual humor, and monetary rewards with a DCM-PEB approach
Source: PLoS One. 2022 Dec 29;17(12):e0279281. doi: 10.1371/journal.pone.0279281 (PMC9799303; doi:10.1371/journal.pone.0279281)
Supplement: S1 File — (DOCX) [file pone.0279281.s001.docx]

**Supplementary information**

**Effective connectivity of the amygdala during the consumption of erotic, sexual humor, and monetary rewards with a DCM-PEB approach**

Yu-Chen Chan^1*^  Tai-Li Chou^2^

^1^Department of Educational Psychology and Counseling, National Tsing Hua University, Hsinchu, Taiwan.

^2^Department of Psychology, National Taiwan University, Taipei, Taiwan

^*^Corresponding author:

Yu-Chen Chan

Department of Educational Psychology and Counseling

National Tsing Hua University

101 Kuang-Fu Road, Section 2

Hsinchu 300044, Taiwan

Tel.: +886-3-5743043

E-mail: ycchan@mx.nthu.edu.tw

Website: https://mx.nthu.edu.tw/~ycchan/

**Contents**

**Part I: Supplementary methods**

1.1 Experimental paradigm

**Part II: Supplementary results**

2.1 Effectivity connectivity for erotic, sexual humor and monetary rewards during the anticipation phase

2.2 Effectivity connectivity for erotic, sexual humor, and monetary rewards during the outcome phase – without hedonic ratings

**Part I: Supplementary methods**

**1.1 Experimental paradigm**

The S1 Fig of the experimental paradigm in the present study was the same as our previous study in Figure 1 using the same dataset [1].


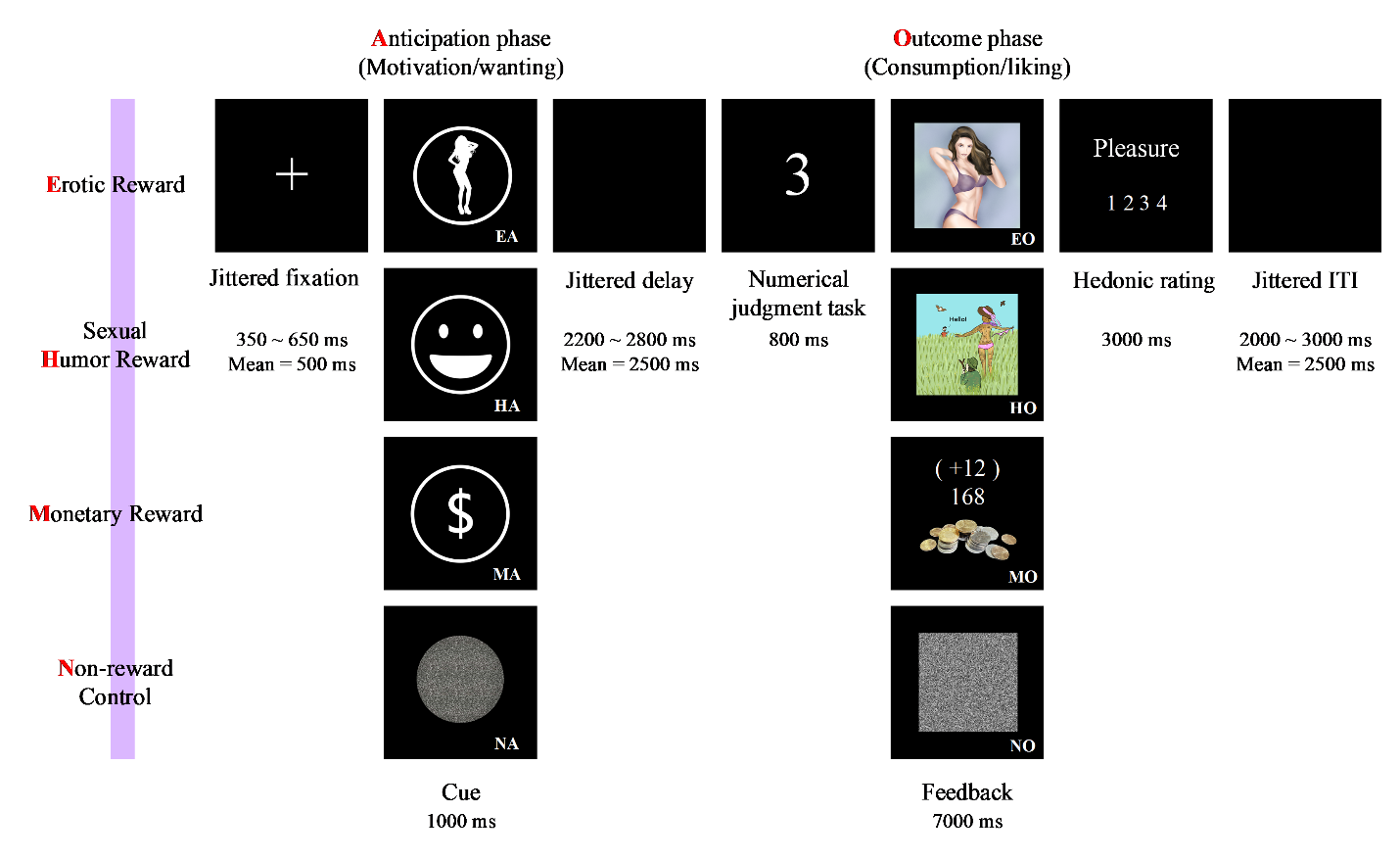


**S1 Fig. Experimental paradigm with four conditions (erotic, sexual humor, monetary, and non-reward) and two phases (anticipation and outcome).** A jittered fixation was presented at the beginning of each trial. Next, a cue for the upcoming reward type was presented for 1000 ms. After a jittered delay period to sustain the anticipatory “wanting” elicited by the cue, participants were given a numerical judgment task. For each trial, a target number that could be either less than 5 (1 to 4) or greater than 5 (6 to 9) was randomly selected and participants had 800 ms to indicate whether the target was less than or greater than 5. Success on the task was rewarded with either an erotic image, a sexual humor cartoon, or a monetary gain (depending on the condition), while a slow or incorrect response led to the non-reward feedback (a matched scrambled image or a gray scrambled image). For the non-reward trials, participants were presented with gray scrambled images regardless of their performance. After each reward outcome, participants had 3000 ms to press a button to give a hedonic rating indicating their level of enjoyment in response to the reward on a 4-point scale (1 for very little pleased; 4 for very highly pleased). Finally, a jittered intertrial interval (ITI) was presented. The erotic and sexual humor images were created by colleagues of the corresponding author in the Cognition, Humor and Affect Neuroscience Laboratory (CHAN LAB). The images in the figure above are similar but not identical to the original images used in the study from which the dataset was taken [1]. They are shown here for illustrative purposes only.

**Part II: Supplementary results**

**2.1 Effectivity connectivity for erotic, sexual humor and monetary rewards during the anticipation phase**

Effective connectivity during the ‘wanting’ or anticipation phase was conducted in the S2 Fig.


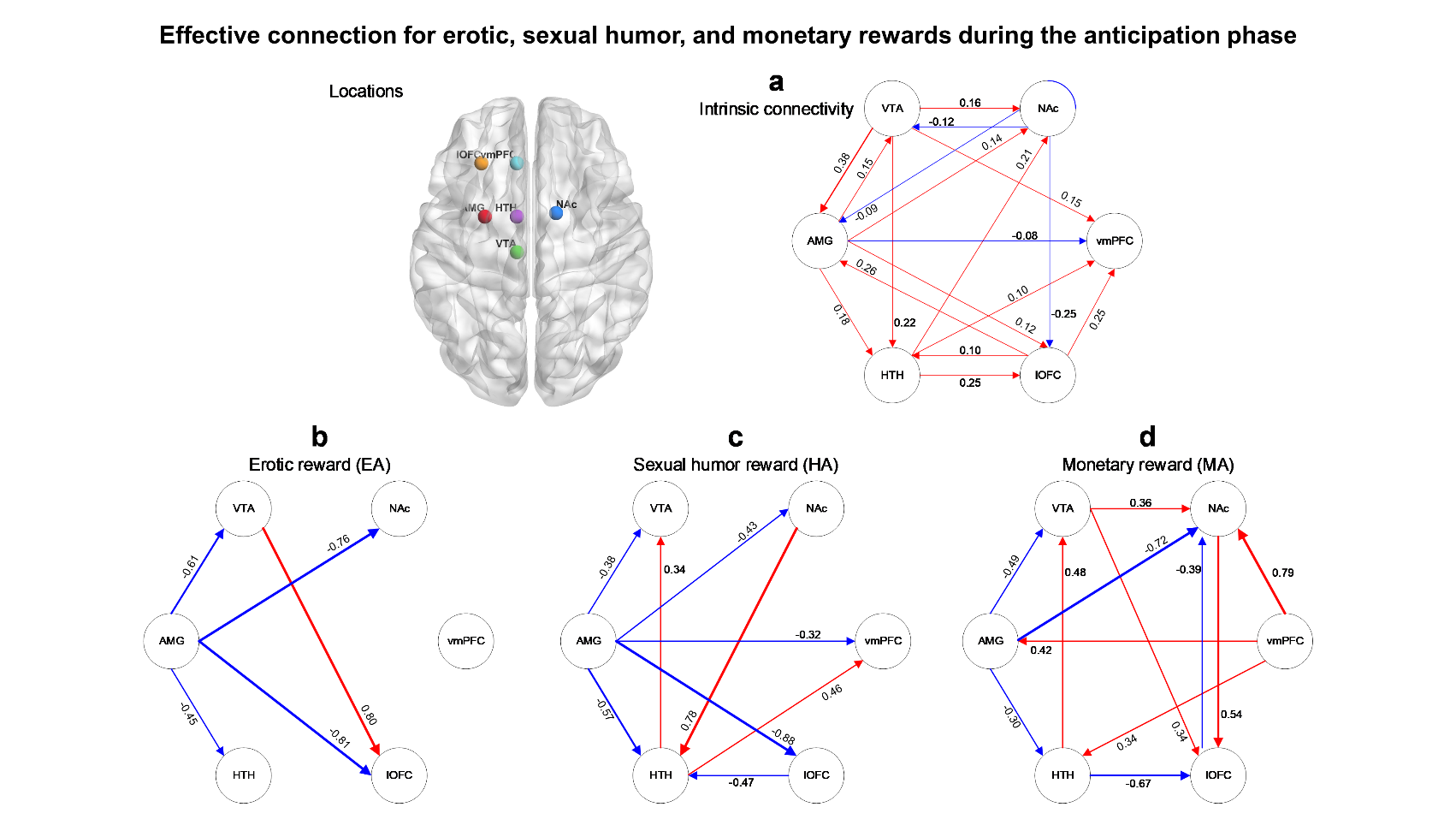


**S2 Fig. Effective connectivity for erotic, sexual humor, and monetary rewards during the anticipation phase.** Results of the DCM analysis are presented as connection weights given by the time constants in Hz for bilinear moderators (B matrix) for which significant effects (at 99% confidence) are indicated by red lines for positive modulatory effects (positive numbers indicate excitatory influence) and blue lines for negative modulatory effects (minus sign indicates inhibitory influences). The numbers indicate the strength of directed connectivity in Hz. The width of the arrows is proportional to the strength of the connectivity in Hz. *A matrix:* (a) Task-independent intrinsic connections. *B matrices:* (b) Modulatory effect for anticipation of erotic stimuli (EA). (c) Modulatory effect of anticipation of sexual humor (HA). (d) Modulatory effect of anticipation of monetary rewards (MA). Self-connections are not displayed in the figure.

**2.2 Effectivity connectivity for erotic, sexual humor, and monetary rewards during the outcome phase – without hedonic ratings**

Effective connectivity during the ‘liking’ or outcome phase was first conducted *without* including the hedonic ratings as covariates. The findings of without participants’ in-scan hedonic ratings provide initial insights during the consumption of sexual and non-sexual rewards in the S3 Fig.


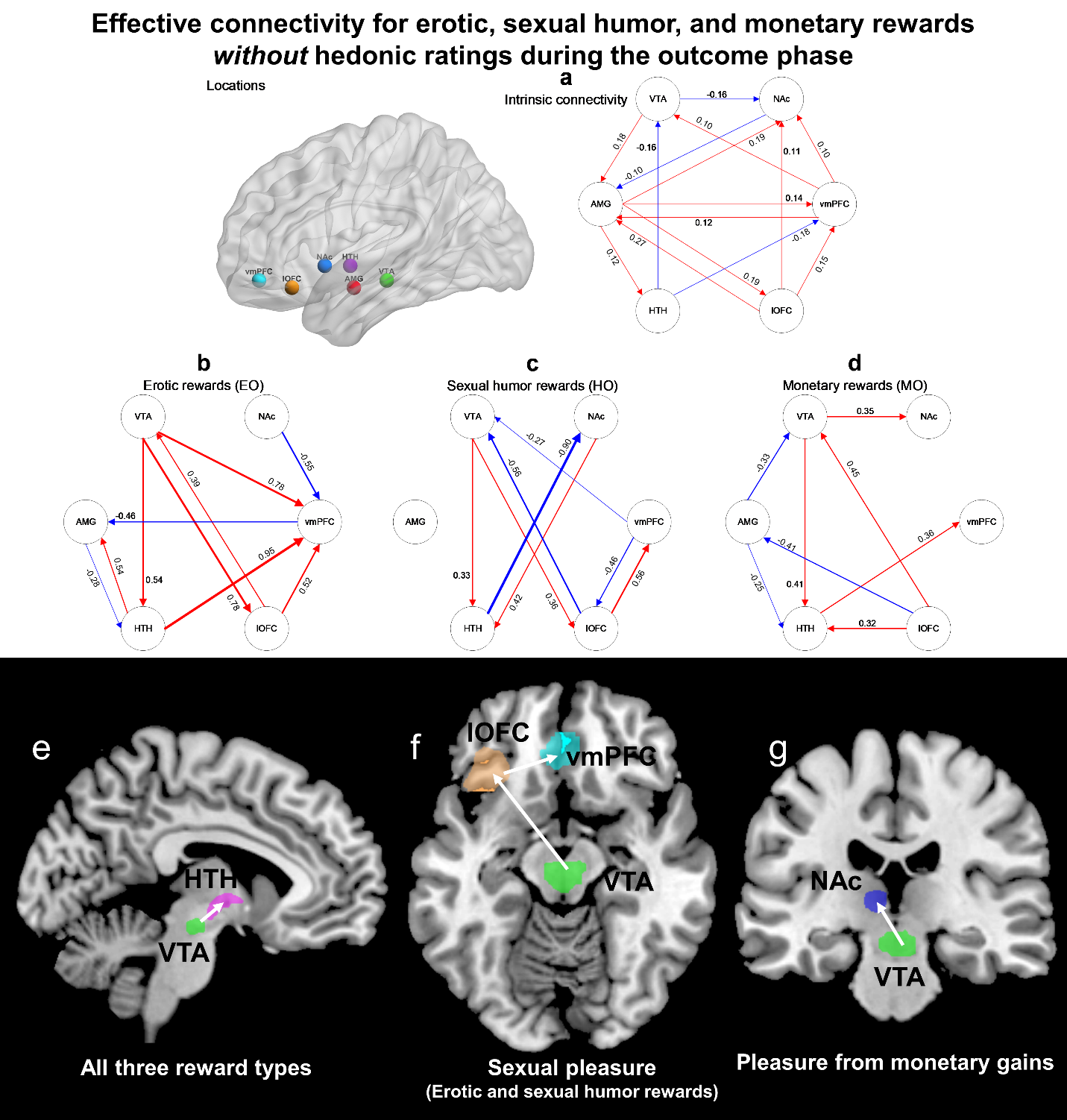


**S3 Fig. DCM results for effective connectivity for the processing of erotic, sexual humor, and monetary rewards *without* hedonic ratings as covariates during the outcome phase.** Values given are time constants (in Hz) for the bilinear influences for which the group posterior mean was significant, with red lines indicating positive modulatory effects and blue lines indicating negative modulatory effects. Significant connections were set at a posterior probability of at least 0.95. Locations stand for the six nodes. (a) Intrinsic connections (A matrix) without ratings as covariates. (b) Modulatory effect of erotic pleasure without ratings as covariates. (c) Modulatory effect of amusement from sexual humor without ratings as covariates. (d) Modulatory effect of pleasure from monetary gains without ratings as covariates. (e) Connections from the VTA to the hypothalamus appear to play a key role in a reward pleasure mechanism for all three reward types. (f) Connections from the VTA to the lOFC and the lOFC to the vmPFC served as a “sexual arousal” mechanism during the appreciation of erotic and sexual humor rewards. (g) Effective connectivity from the VTA to the NAc was identified for both the anticipation and consumption/enjoyment of monetary gains. Self-connections are not displayed in the figure.

**Reference**

1. Chan, YC, Hsu, WC, Chou, TL. Differential neural substrates for responding to monetary, sexual humor, and erotic rewards. Biol Psychol. 2022; 172:108385. <https://doi.org/10.1016/j.biopsycho.2022.108385> PMID: 35777520
